# Balance Assessment Using Gamified Digital Technology in Community-Dwelling Older Adults: Mixed Methods Validation Study and Randomized Controlled Trial

**DOI:** 10.2196/80747

**Published:** 2026-04-15

**Authors:** Jianan Zhao, Yaqin Xia, Yahui Zhang, Jihong Yu, Chen Chu

**Affiliations:** 1 College of Fashion and Design, Donghua University, Yanan Road #1448, Shanghai, 200240, China; 2 School of Design, Shanghai Jiao Tong University, Dongchuan Road 800#, Shanghai, 200240, China, 86 18200484800

**Keywords:** digital health, gamification, older adults, balance testing, user engagement

## Abstract

**Background:**

Falls cause injury and mortality among older adults, necessitating reliable, scalable, engaging balance assessment tools to use in community settings. Traditional clinician-administered assessments like the Brief Balance Evaluation Systems Test (Brief-BESTest) are limited by subjectivity and accessibility constraints. Computer vision–based digitalization combined with gamification may address these limitations; yet, validation evidence remains limited.

**Objective:**

We (1) digitalized and validated a computer vision–based Brief-BESTest against clinician scoring and (2) investigated whether a gamified interface improves older adults’ user experience during balance assessment, without compromising assessment performance.

**Methods:**

This mixed methods study comprised (1) a concurrent validity substudy in a convenience subsample (n=10) and (2) a parallel-group randomized controlled trial (RCT; n=30) with 1:1 allocation. Participants were community-dwelling older adults aged ≥60 years recruited from Hongqi Community, Shanghai, through community announcements and health care worker referrals. Phase 1 (n=10; mean age 64.9, SD 2.76 years) evaluated concurrent validity of a computer vision-based digitalized Brief-BESTest using OpenPose skeletal tracking (Carnegie Mellon University Perceptual Computing Lab; Logitech Brio 4K webcam, 27-inch touchscreen) against the clinician-administered version. Phase 2 (n=30; mean age 66.7, SD 3.93 years) used a parallel-group RCT with 1:1 coin-flip allocation. Primary outcome measures include perceived exertion (Borg Rating of Perceived Exertion scale 6‐20), intrinsic motivation (Intrinsic Motivation Inventory 7-point Likert, including interest and enjoyment, perceived competence, and pressure and tension subscales), and intention to continue use (7-point Likert scale). Semistructured interviews (mean 4.8 minutes) assessed engagement factors. Data collection occurred in a controlled indoor setting with safety railings.

**Results:**

Phase 1 demonstrated excellent intrasession reliability (intraclass correlation coefficient=0.89‐0.92) and strong concurrent validity (Spearman ρ=0.91; 95% CI 0.68‐0.98; *P*<.01), with no significant mean difference (MD; paired *t* test: MD 0.23; *P*=.77; *d*=−0.07). In phase 2, Gamified Digital Balance Assessment (GDBA) users reported significantly lower perceived exertion (Mann-Whitney *U*: MD −2.67; 95% CI −4.60 to −0.74; *P*=.01; *d*=−1.08), higher enjoyment (MD 1.53; *P*=.009; *d*=1.17), higher perceived competence (MD 1.14; *P*=.02; *d*=0.89), and higher intention to continue use (MD 1.66; *P*=.001; *d*=1.25). Pressure and tension (*P*=.09; *d*=0.63) showed no significant difference. Thematic analysis (Cohen κ=0.68) identified 2 themes: motivational rewards (80% cited real-time feedback) and perceived usability (87% emphasized avatar demonstrations).

**Conclusions:**

This study validated a computer vision–based digital Brief-BESTest and experimentally tested a gamified interface for balance assessment in community-dwelling older adults. Unlike prior work focused largely on single-task digital tests or nongamified interfaces, the GDBA integrates comprehensive, clinically grounded balance assessment with evidence-based gamification tailored to older users. These findings advance digital geriatric assessment by demonstrating that gamified designs can enhance motivation, perceived competence, and tolerability of testing without sacrificing measurement quality. If replicated in longitudinal and real-world settings, such systems could provide scalable, low-cost tools for routine fall-risk screening, self-monitoring, and targeted preventive interventions in community and primary-care environments.

## Introduction

Falls are a leading cause of injury, disability, and mortality among older adults, with approximately one-third of community-dwelling individuals aged 65 years or older experiencing a fall each year [1]. A recent comprehensive systematic review and meta-analysis estimated the global prevalence of falls in older adults at 26.5%, underscoring that roughly one in four older adults falls annually [2]. In China, the burden is amplified by a rapidly aging population, which is projected to exceed 400 million people aged 60 years and older by 2040 [3]. This demographic trend increases both absolute numbers of fall events and the potential demand for community-based prevention and monitoring services [4]. Effective fall prevention strategies are urgently needed to address the rising health care demands of older populations, particularly in community settings where access to regular clinical assessment is limited [5,6].

Routine balance assessment is a cornerstone of fall prevention, enabling early identification of postural instability and guiding timely intervention. However, conventional balance assessments—such as the Brief Balance Evaluation Systems Test (Brief-BESTest)—are typically conducted in clinical environments by trained professionals, limiting accessibility for independently living older adults, especially in underserved or rural areas [7]. Moreover, clinic-based assessments are time- and resource-intensive, and many older adults receive only episodic screening; this gap motivates interest in remote and automated assessments that preserve clinical validity while increasing reach [8]. Advances in computer vision technologies present an opportunity to overcome these limitations by facilitating contactless, automated assessments that can be delivered outside clinical settings [9].

Existing digital balance testing systems frequently rely on assessments such as the Timed Up and Go (TUG) Test [10] or the Sit-to-Stand Test [11], which primarily evaluate isolated or simple movements. While these tools are useful, they may lack the multidimensional scope needed for a comprehensive evaluation of balance [12]. In contrast, the Brief-BESTest is a validated and concise clinical tool that assesses 6 key domains of postural control—biomechanical constraints, stability limits, anticipatory adjustments, reactive postural responses, sensory orientation, and stability in gait—making it well-suited for a more holistic assessment of balance in older adults [13,14]. Validation studies support the construct validity of the BESTest family (BESTest, mini-BESTest, and Brief-BESTest) in community-dwelling older adults and demonstrate that these tools discriminate fallers from nonfallers and correlate with established functional measures, which argues for translating the Brief-BESTest’s multidimensional structure into a digital format rather than relying solely on single-metric tests [15]. Nevertheless, there are relatively few peer-reviewed implementations that fully translate a multidomain instrument like the Brief-BESTest into a markerless computer-vision pipeline suitable for unsupervised community use; existing digital systems more commonly implement TUG, sit-to-stand, or gait-only assessments [16].

In addition to technical feasibility, sustaining engagement among older users is essential. Gamification—the integration of game design elements such as feedback, rewards, and interactive visuals—has been shown to enhance motivation and adherence in digital health applications [17,18]. A systematic review of gamification for older adults found overall positive effects on engagement, enjoyment, and adherence when gameful elements are appropriately tailored; however, the review also highlights heterogeneity in design choices and a need for stronger evidence linking gamification to objective clinical outcomes in older populations [19]. Recent evidence suggests that well-designed gamified interventions can reduce perceived effort and increase autonomous motivation in older adults performing physical assessments [20]. For older adults, interactive systems may reduce anxiety, improve perceived competence, and encourage repeated use of assessment tools [21,22]. Specifically, real-time visual feedback and avatar-based demonstrations have been identified as key design elements that support self-efficacy and reduce cognitive load during technology-mediated assessments [23]. Yet, little is known about the impact of gamifying comprehensive balance assessments like the Brief-BESTest on user experience and continued use intentions [24].

This study addresses two critical gaps: (1) the absence of a valid and reliable, computer vision–based version of the Brief-BESTest suitable for community deployment, and (2) limited understanding of the role of gamification in enhancing older adults’ engagement with digital balance assessments. We present the development and evaluation of the Gamified Digital Balance Assessment (GDBA), which integrates automated Brief-BESTest scoring with gamified interface design. Through a 2-phase mixed methods study, we tested the following objectives and hypotheses:

1. To validate the digitalized Brief-BESTest against the clinician-administered version, hypothesizing strong concurrent validity and reliability.
2. To evaluate the impact of gamification on user experience, hypothesizing that the GDBA would result in lower perceived exertion and higher intrinsic motivation compared to the nongamified version.

## Methods

### Patient and Public Involvement

No patients or members of the public were formally involved in setting the research questions, outcome measures, intervention design, or recruitment procedures for this trial. A total of 30 community-dwelling older adults participated in the randomized controlled trial. A convenience subsample of 10 participants contributed to the concurrent validity substudy. Participants served only as study participants and were not involved in study governance or dissemination planning.

### Trial Design

This 2-phase study was conducted at the Hongqi Community Center in Shanghai in April-May 2025. Participant enrollment for the dataset reported here took place between April 20 and May 20, 2025. It used a sequential 2-phase mixed methods design. Phase 1 used a within-subjects concurrent validity design with repeated measures. Phase 2 used a parallel-group randomized controlled trial design with 1:1 allocation, supplemented by qualitative semistructured interviews that carefully follow the CONSORT (Consolidated Standards of Reporting Trials) guideline (Checklist 1) [25]. The Phase 2 randomized controlled trial was retrospectively registered at ClinicalTrials.gov (NCT06958653) after enrollment began (registration date: July 17, 2025). The study team initially categorized the Phase 2 component as a low-risk formative evaluation focused on user experience of a digital assessment interface and did not recognize that any randomized allocation of participants requires prospective trial registration under International Committee of Medical Journal Editors (ICMJE)/JMIR policy. Once this requirement was identified during manuscript preparation, we registered the trial and are updating the registry record to transparently reflect the outcomes and their timing as collected and reported in this manuscript. This research collected written informed consent from all participants and was reported following the Journal Article Reporting Standards (JARS) for mixed methods research [26].

### Participant Characteristics, Sampling Procedures, and Selection Criteria

Community-dwelling older adults were recruited offline from the Hongqi Community in Shanghai through community announcements, poster displays at the community center, and referrals from community health care workers. Interested individuals were screened for eligibility via telephone by a trained research assistant using a standardized eligibility checklist. Eligible participants were then invited for an in-person informed consent session prior to enrollment. Inclusion criteria for both phases were (1) age ≥60 years, (2) ability to stand unassisted for 30 seconds and walk 10 m independently, and (3) no acute illness at the time of testing. Exclusion criteria included (1) diagnosed neurological conditions affecting balance, (2) uncorrected visual or auditory impairments, (3) recent fall requiring medical attention, and (4) use of medications known to affect postural stability.

### Sample Size, Power, and Precision

For Phase 1, a sample of 10 participants was selected for reliability studies. For Phase 2, sample size was determined a priori using G*Power 3.1 software (Heinrich Heine University Düsseldorf). Assuming a 2-tailed independent *t* test, α=.05, power (1-β)=.80, and a medium-to-large effect size (Cohen *d*=0.55) based on prior gamification studies in older adults [21], the required sample size was calculated as n=28. We recruited 30 participants (15 per group) to account for potential dropouts, though none occurred.

Systematic assessment of harms was not a primary objective of this study given the low-risk nature of balance testing in a controlled environment with safety measures. However, we monitored for adverse events during all testing sessions. Potential harms were defined as (1) falls or near-falls during balance tasks and (2) musculoskeletal discomfort or pain exacerbation. All testing was conducted with safety railings present and a trained researcher monitoring participants. No adverse events occurred during the study period. Participants were instructed to stop immediately if they experienced any discomfort, and emergency protocols were established in consultation with local health care providers.

### Product Development

#### Digitalized Brief-BESTest Design

The digitalized Brief-BESTest was designed to digitize and automate the Brief-BESTest. While the traditional clinician-administered Brief-BESTest relies on subjective scoring, the digitalized Brief-BESTest enables self-guided assessments with automated, objective scoring, improving accessibility in community and home settings.

The system uses OpenPose, an open-source pose estimation framework validated for human movement analysis, to capture skeletal data via a standard 2D RGB camera. OpenPose was selected based on evidence demonstrating its accuracy in tracking older adult movements in clinical contexts [27] and its suitability for noninvasive, contactless assessment without wearable sensors. The system tracks 17 anatomical landmarks (eg, nose, neck, shoulders, elbows, wrists, hips, knees, and ankles) at 30 frames per second.

Ten joint angles relevant to static and dynamic postural tasks (eg, hip flexion, knee flexion, ankle dorsiflexion, and trunk inclination) are computed from the landmark coordinates. The torso vector is defined from the neck landmark to the midpoint between the left and right hip landmarks, serving as a reference axis for postural alignment calculations.

To convert pixel-based coordinates into metric units (centimeters), the system uses participants’ self-reported height adjusted by anthropometric correction factors derived from ISO 7250‐1 international standards. These correction factors (10.77 cm for men, 10.06 cm for women) represent the average vertical distance from the top of the head to the C7 vertebra (neck landmark detected by OpenPose), as established in large-scale anthropometric databases. These values have been validated for use in Chinese adult populations [28]. The corrected height is then used to normalize skeletal dimensions, enabling calculation of joint angles and postural parameters in standardized metric units.

The digitalized Brief-BESTest replicates all 8 tasks from the validated Brief-BESTest: (1) lift leg to the side, (2) lift 2 arms, (3) stand on one leg-left, (4) stand on one leg-right, (5) take a step to the left, (6) take a step to the right, (7) stance with eyes closed, and (8) up and go. These 8 tasks cover all 6 domains of the original Brief-BESTest, including biomechanical constraints, stability limits/verticality, anticipatory postural adjustments, postural responses, sensory orientation, and stability in gait.

Key kinematic parameters include (1) torso angle deviation from vertical (quantifying postural sway), (2) joint angles (hip, knee, ankle flexion/extension, and abduction/adduction), (3) center of mass displacement (estimated via pelvis midpoint), (4) temporal metrics (stance duration and time to stabilization), and (5) spatial metrics (reach distance, step length, and base of support width).

Representative scoring examples include the following: for single-leg stance (Items 3‐4), participants scoring 3 points maintain stance ≥20 seconds with postural sway <3° and hands on hips, while those scoring 0 points cannot maintain stance ≥2 seconds. For functional reach forward (Item 2), score 3 requires ≥32 cm forward reach without heel lift or trunk rotation, while score 0 indicates <3 cm displacement or loss of balance. For the TUG test (Item 8), score 3 requires completion in ≤11 seconds with trunk sway <5° during gait, while score 0 indicates >11 seconds with significant imbalance (trunk sway >8°, stumbles, or requiring assistance).

The total balance score (0‐24) is calculated as the sum across all eight items, with established cutoffs for fall risk interpretation: 0‐12 (high risk), 13‐18 (moderate risk), and 19‐24 (low risk).

#### GDBA Design

**Figure 1.**
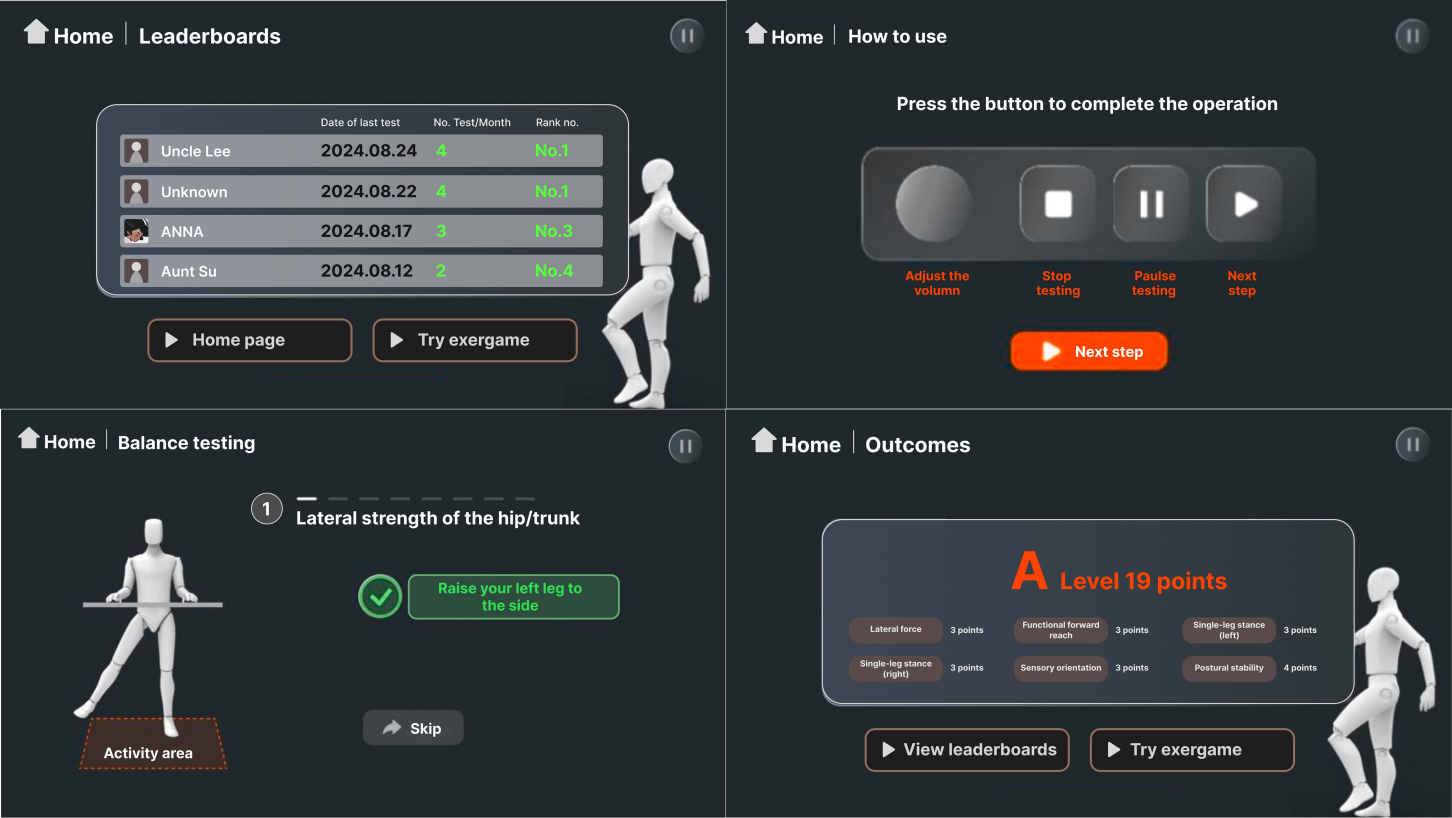
User interface examples of the Gamified Digital Balance Assessment (GDBA) system showing the task instruction screen with animated avatar demonstration, real-time performance feedback during balance task execution, and postassessment summary with score visualization and leaderboard. Hongqi Community Center, Shanghai, China, 2025.

The GDBA builds upon the digitalized Brief-BESTest by incorporating evidence-based gamification elements designed to enhance motivation and engagement among older adults. The gamification design was guided by self-determination theory [29], which posits that autonomy, competence, and relatedness are key drivers of intrinsic motivation, and by recent systematic reviews on gamification for older adult health interventions [23,30]. The examples of the interface are presented in Figure 1. Several core game mechanics were implemented.

- Points and scoring: each balance task awards 0‐3 points based on performance, with real-time point accumulation displayed prominently. Achieving higher scores unlocks encouraging messages (eg, “Excellent balance!”).
- Animated avatars: a 3D avatar demonstrates each task before the participant performs it, showing ideal posture and movement. Avatar design was built based on the shapely human figure without specific age references to enhance relatedness.
- Progress visualization: a dynamic progress bar shows overall completion status, and a colored time count timer helps participants pace themselves during timed tasks.
- Performance summary report: upon completion, participants receive a comprehensive report including total score with fall risk category, domain-specific subscores, task-level feedback, and personalized training recommendations.
- Optional leaderboard: an anonymized leaderboard displays recent user rankings (initials and scores only) with emphasis on personal progress over social comparison. Participants can opt out during initial setup.
- Multimodal accessibility design: the interface features high-contrast colors (black background, orange, and green highlights), large fonts (≥24 point), voice narration in Mandarin (120 words per min), and simplified navigation with large physical buttons (≥100×100 pixels) to accommodate age-related sensory and motor changes.
- Difficulty levels and progression (under development): although not implemented in this study, the system is designed to support adaptive difficulty training sessions (eg, unlocking advanced balance exercises after achieving baseline competence). These features are planned for future versions to support long-term engagement.
- Hardware setup: the assessment environment comprises a 1 m×3 m zone with safety railings (height: 90 cm) on all sides and a centrally placed 1 m×1 m ethylene-vinyl acetate (EVA) foam pad (10 cm thick, 35D density) for sensory orientation tasks. Hardware includes a Logitech Brio 4K webcam mounted 2.5 m from the assessment center; a 27-inch LCD touchscreen display positioned at eye level (adjustable 1.2‐1.7 m); an embedded computer (Intel i7, 16 GB RAM, NVIDIA GTX 1650 GPU) running custom Python software (Python Software Foundation; OpenPose for tracking, NumPy/SciPy for kinematics, Figma for interface); and a detachable ergonomic control console with 3 large tactile buttons designed per Chinese anthropometric standards (GB 10000‐88). All components are optimized for older adult users aged >60 years.

### Study Procedure and Data Diagnostics

The study was conducted in 2 sequential phases to evaluate the reliability and user experience of a GDBA tailored for community-dwelling older adults.

#### Phase 1: Reliability of Digitalized Brief-BESTest Assessment

In the first phase, participants performed a single balance assessment session, during which both the clinician-administered Brief-BESTest and the digitalized Brief-BESTest were scored concurrently. This approach enabled direct comparison between clinical and automated assessments under identical task conditions.

Testing was conducted in a controlled indoor setting featuring a 1 m×1 m, 10-cm-thick EVA foam mat (35D density) and safety handrails on 3 sides. Prior to the assessment, participants completed a baseline questionnaire collecting demographic data (age and sex), anthropometric measurements (height and weight), and fall history (past 12 months).

During the assessment, a certified physical therapist delivered standardized verbal instructions and rated each task using the validated Brief-BESTest rubric (maximum score=24). Simultaneously, the digitalized Brief-BESTest system recorded participants’ movements using a monocular 4K camera and calculated scores via an algorithm that mirrors the original scoring criteria. The torso and joint movements were analyzed in real time, and balance scores were automatically computed.

To evaluate interrater reliability, a second trained clinician independently rated 20% of the sample. Both raters had more than 5 years of clinical experience and completed standardized training on Brief-BESTest administration prior to the study. This concurrent scoring design ensured consistent task execution while enabling evaluation of intermethod reliability of the automated system’s scoring against expert clinician judgment.

Potential Assessor Bias Mitigation: while the primary clinician-administered assessments were conducted by a single certified physiotherapist to ensure consistency in instruction delivery, we acknowledge this may introduce assessor-specific bias (eg, subjective interpretation of borderline performance). To mitigate this limitation, we (1) used a second independent rater for 20% of cases to establish interrater reliability (intraclass correlation coefficient [ICC]=0.94; 95% CI 0.85‐0.98), (2) ensured the primary rater followed the standardized Brief-BESTest manual verbatim, and (3) video-recorded all sessions to allow for post hoc verification. Future studies should use multiple assessors across sessions to further minimize individual rater effects.

#### Phase 2: Impact of GDBA on User Experience

The second phase involved a parallel group randomized controlled trial (RCT) to assess the impact of gamification on user experience.

**Figure 2.**
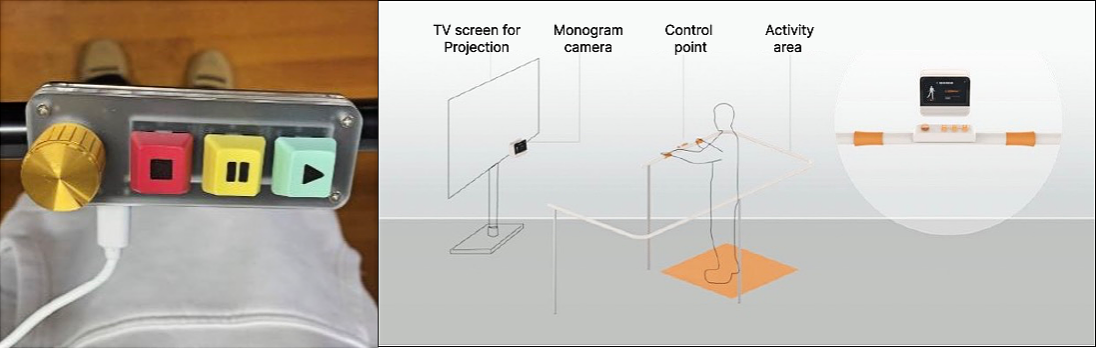
Experimental setup for the evaluation of the Gamified Digital Balance Assessment (GDBA) in community-dwelling older adults (N=30, aged >60 years), including a 1 m×3 m assessment zone with safety railings and an ethylene-vinyl acetate (EVA) foam mat, a 4K camera and display system for motion tracking and visual feedback, and an ergonomically designed user control console. Hongqi Community Center, Shanghai, China, 2025.

After informed consent, participants were allocated using a simple coin-flip method (heads=control, tails=experimental) to either the control group (uses digitalized Brief-BESTest) or the experimental group (uses GDBA) through a simple coin-randomization method by a blinded researcher. Questionnaires that assessed their demographic information, fall history, and balance confidence were used. Testing was conducted in a 1 m× 3 m evaluation zone equipped with front, side, and rear safety railings and a centrally placed EVA foam pad (identical to Phase 1). The DBTS system included a display screen, a Logitech Brio 4K webcam (30 fps) for motion tracking, and a built-in speaker for voice prompts. A detachable, ergonomically designed user console—compliant with Chinese anthropometric standards—was mounted on the front railing for interface navigation (see Figure 2).

In the control group, participants performed balance tasks following prerecorded verbal instructions from a certified physical therapist. In the experimental group, tasks were presented via the GDBA interface, which included animated avatars, voice guidance, progress indicators, and real-time performance feedback. Each participant completed one practice trial per task to minimize learning effects, followed by the formal assessment. A 2-minute seated rest period was provided between tasks to reduce fatigue.

Immediately following the assessment, participants completed self-report measures on perceived exertion, intrinsic motivation, and intention for continued use. They then participated in a brief semistructured interview exploring their perceptions of system usability and engagement. All participants (n=30) completed individual semistructured interviews (mean duration=4.8 min, range=3‐7 min) immediately post assessment. The interviewer was aware of group assignment but blinded to quantitative scores. All interviews were audio-recorded and transcribed verbatim in Mandarin within 48 hours. Transcripts were verified for accuracy by the primary interviewer. Participants received a nominal compensation (US $10 equivalent) upon study completion.

Each participant attended a single testing session lasting approximately 25‐35 minutes with no interim analyses, which included informed consent, a baseline questionnaire, a balance assessment, postassessment questionnaires, and qualitative interviews. The participants can quit the experiment whenever they want. This cross-sectional design enabled evaluation of immediate user experience but did not assess longitudinal adherence or learning effects over multiple sessions.

### Outcome Measures and Covariates

#### Baseline Variable

Balance ability was assessed using the Brief-BESTest, a validated clinical tool comprising 8 tasks across 6 domains of postural control, including biomechanical constraints, stability limits/verticality, anticipatory postural adjustments, postural responses, sensory orientation, and stability in gait [13]. Each task is scored on a 0‐3 scale (0=inaccurate performance, 3=normal performance), yielding a total score of 0‐24, with higher scores indicating better balance.

Balance confidence was assessed as a baseline variable using the Activities-specific Balance Confidence (ABC) scale (0%‐100%); for reporting consistency, ABC scores were normalized to a 0‐1 scale by dividing by 100 and were collected immediately after the assessment session. Higher scores indicate greater balance confidence.

#### User Experience

Perceived physical exertion was measured using the Borg Rating of Perceived Exertion (RPE) Scale, ranging from 6 (“no exertion”) to 20 (“maximal exertion”) [31]. Participants verbally reported their RPE immediately after completing all balance tasks to reflect overall physical demand and fatigue during the assessment. This measure provided insight into the tolerability and physical burden of the assessment procedures.

Motivational engagement was evaluated using the Intrinsic Motivation Inventory (IMI), a validated tool using a 7-point Likert scale (1=“not at all true,” 7=“very true”) [32]. Three subscales were analyzed, including interest/enjoyment (assesses task engagement and inherent enjoyment of the activity), perceived competence (measures self-perceived ability and confidence in performing the tasks), and pressure/tension (evaluates task-related stress and anxiety). Subscale scores were calculated as the mean of item responses, with higher scores indicating greater enjoyment, competence, or pressure, respectively. The IMI has demonstrated good internal consistency and construct validity in older adult populations [33].

Intention to continue use was assessed through both quantitative and qualitative methods. Quantitatively, participants rated their likelihood of using the system again on a single-item 7-point Likert scale (1=“very unlikely,” 7=“very likely”) immediately following the assessment. Qualitatively, semistructured interviews explored factors influencing future use intentions. Interview questions included, “Would you consider using this system regularly for balance checking? Why or why not?” and “What features would encourage you to use this system more often?” Interviews lasted 3‐5 minutes, were audio-recorded with permission, transcribed verbatim, and analyzed using the 6-phase thematic analysis framework.

### Training and Reliability of Data Collectors

Data collection was conducted by trained research personnel who completed a standardized 4-hour training session on Brief-BESTest administration, equipment operation, and participant interaction protocols. Training included video-based instruction, practice sessions with pilot participants, and competency assessment by the principal investigator. Interrater reliability was established by having 2 certified physiotherapists (both with >3 years’ experience in geriatric assessment) independently score 20% (2/10) of Phase 1 participants, achieving excellent agreement. The digitalized Brief-BESTest system underwent technical validation prior to data collection, including calibration testing with known reference objects to verify measurement accuracy. For qualitative interviews, the primary interviewer (JZ) received training in semistructured interview techniques and motivational interviewing principles. Interview fidelity was maintained through use of a standardized interview guide and audio recording of all sessions for quality review.

### Psychometrics

Reliability in this sample: internal consistency was assessed for all multi-item scales in this sample (n=30, Phase 2). The IMI subscales demonstrated good internal consistency, including interest and enjoyment (Cronbach α=.87; 95% CI 0.76‐0.93), perceived competence (α=.84; 95% CI 0.71‐0.91), and pressure and tension (α=.79; 95% CI 0.63‐0.88). The Brief-BESTest total score showed excellent internal consistency in this current sample (α=.91; 95% CI 0.83‐0.95). Test-retest reliability was not assessed in this study due to the single-session design. However, the digitalized version demonstrated excellent intrasession reliability (ICC=0.89‐0.92) across multiple automated scoring iterations of the same video recordings.

Convergent validity: convergent validity of the digitalized Brief-BESTest was supported by strong correlation with the clinician-administered version (Spearman ρ=0.91; *P*<.01), suggesting both methods measure the same construct.

### Statistical Methods

All quantitative analyses were conducted using SPSS (IBM Corp). To assess the validity of the digitalized Brief-BESTest, balance scores from the clinician-administered and algorithm-generated assessments were compared using paired *t* tests or Wilcoxon signed-rank tests, depending on normality. Effect sizes were calculated using Cohen d. Interrater reliability for the clinician-administered Brief-BESTest was assessed on 20% of cases scored by 2 clinicians using the intraclass correlation coefficient (ICC, 2-way mixed model, absolute agreement). ICC values >0.75 were considered excellent, 0.40‐0.75 moderate to good, and <0.40 poor. Convergent validity was examined via Spearman correlation (ρ).

In the second phase, nonparametric tests (Mann–Whitney *U*) were used to compare user experience outcomes—perceived exertion, intrinsic motivation, and intention to continue use—between the experimental group and the control group. IMI subscales (interest/enjoyment, perceived competence, and pressure/tension) were analyzed separately. Significant effects were reported with Cohen *d*. Qualitative interview data were analyzed thematically [34] by the primary interviewer (JZ, female, PhD in digital health; CC, male, MSc in health informatics). The coding team included 2 independent analysts. Neither had prior relationships with participants. Reflexivity measures included maintaining research journals and weekly debriefing sessions during the 3-week analysis period. Analysis followed six phases: (1) familiarization, both coders independently read all 30 transcripts twice; (2) generating initial codes, line-by-line coding in NVivo 14 (Lumivero; Release 1.7.1) produced 156 initial codes; (3) searching for themes, related codes were grouped into 8 candidate themes through iterative discussion; (4) reviewing themes, candidate themes were reviewed against the dataset for internal coherence and external distinction; (5) defining themes, final themes were refined with clear operational definitions; and (6) producing the report, exemplar quotes were selected to illustrate each theme. Intercoder agreement was moderate (Cohen κ=0.68); all discrepancies were resolved through consensus discussion. Data saturation was assessed iteratively; no new themes emerged after interview 25 (experimental group: interview 13; control group: interview 12), suggesting thematic saturation was achieved. Member checking was not conducted due to the brief time frame and immediate postassessment interview design; however, findings were triangulated with quantitative data to enhance credibility.

### Ethical Considerations

This study was approved by the Institutional Review Board of Shanghai Jiao Tong University (approval number: H20240601I, approved on January 12, 2024). The randomized controlled trial was retrospectively registered at ClinicalTrials.gov (NCT06958653) on July 17, 2025, after enrollment began on April 20, 2025. The delay occurred because the study team initially categorized the randomized component as a low-risk formative evaluation of user experience for a digital assessment interface and did not recognize that randomized allocation requires prospective registration under ICMJE/JMIR policy; once identified during manuscript preparation, the ClinicalTrials.gov record has been corrected/updated to reflect the RCT sample size (n=30) and the substudy sample size (n=10), to designate user experience as the primary outcome domain, and to list the ABC scale as a baseline characteristic rather than an outcome, with the primary outcomes being user experience measures (perceived exertion, intrinsic motivation, and intention to continue use).

All procedures were conducted in accordance with the Declaration of Helsinki. All participants provided written informed consent after receiving detailed verbal and written explanations of the study purpose, procedures, potential risks, and benefits. All participant data were deidentified using unique numeric codes. Video recordings captured during balance assessments contained only skeletal joint position data and did not record identifiable facial features or other personal characteristics. All images included in the manuscript are representative schematics or composite visualizations created from anonymized skeletal data; no actual participant photographs are shown. The interface screenshots and environmental setup photos were taken in empty testing environments without participants present, ensuring no individual identification is possible. All participants were informed that no identifiable images would be captured or published. Participants received 70 Chinese Yuan (approximately US $10) as compensation for their time upon completion of the study session. To minimize fall risk during balance assessments, all testing was conducted in a designated area equipped with safety railings on 3 sides.

## Results

### Phase 1

In the first phase, 10 older adults (mean age 64.9, SD 2.76 years; 5 men, 5 women) participated; 5 were excluded due to recent falls requiring medical attention (n=4) and declined to participate (n=1). Of these, 30% (3/10) reported a prior history of falls. The mean BMI was 26.7 kg/m² (SD 3.1).

Balance performance scores from the clinician-administered Brief-BESTest and the digitalized Brief-BESTest were 14.3 (SD 3.24) and 14.53 (SD 3.03), respectively. A paired-sample *t* test revealed no significant difference between the 2 assessments (*P*=.77; Cohen *d*=–0.07), indicating strong similarity in 2 sets of results.

Absolute reliability, assessed via the SE of measurement, demonstrated low variability in scores. Relative reliability was reasonable across all 6 Brief-BESTest domains for both assessment methods. ICCs for the clinician-administered version ranged from 0.84 to 0.96 (ICC=0.92; 95% CI 0.84‐0.96). For the digitalized version, ICCs ranged from 0.78 to 0.94 (ICC=0.89; 95% CI 0.78‐0.94). Mean differences (MDs) between the 2 test scores ranged from 0.2 to 0.4 points, indicating high consistency.

**Figure 3.**
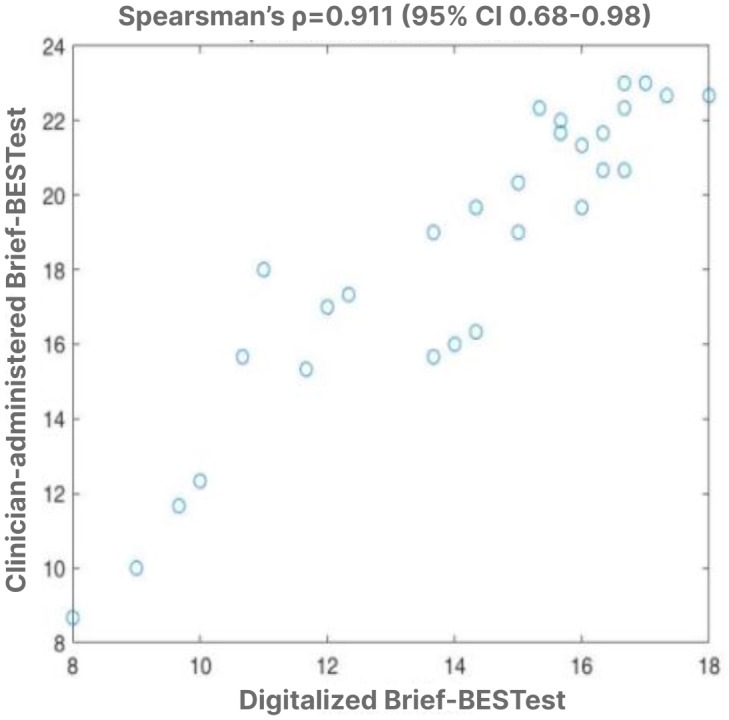
Correlation between balance scores obtained from the clinician-administered Brief Balance Evaluation Systems Test (Brief-BESTest) and the digitalized Brief-BESTest in community-dwelling older adults (n=10; mean age 64.9, SD 2.76 years; 50% female; Shanghai, China, April 2025). Spearman ρ=0.91 (95% CI 0.68–0.98).

Convergent validity analysis, as shown in Figure 3, showed a strong and significant positive correlation between the 2 assessment methods (Spearman ρ=0.91; *P*<.01), confirming that the digitalized Brief-BESTest provides results comparable to the clinician-administered Brief-BESTest.

### Phase 2

**Figure 4.**
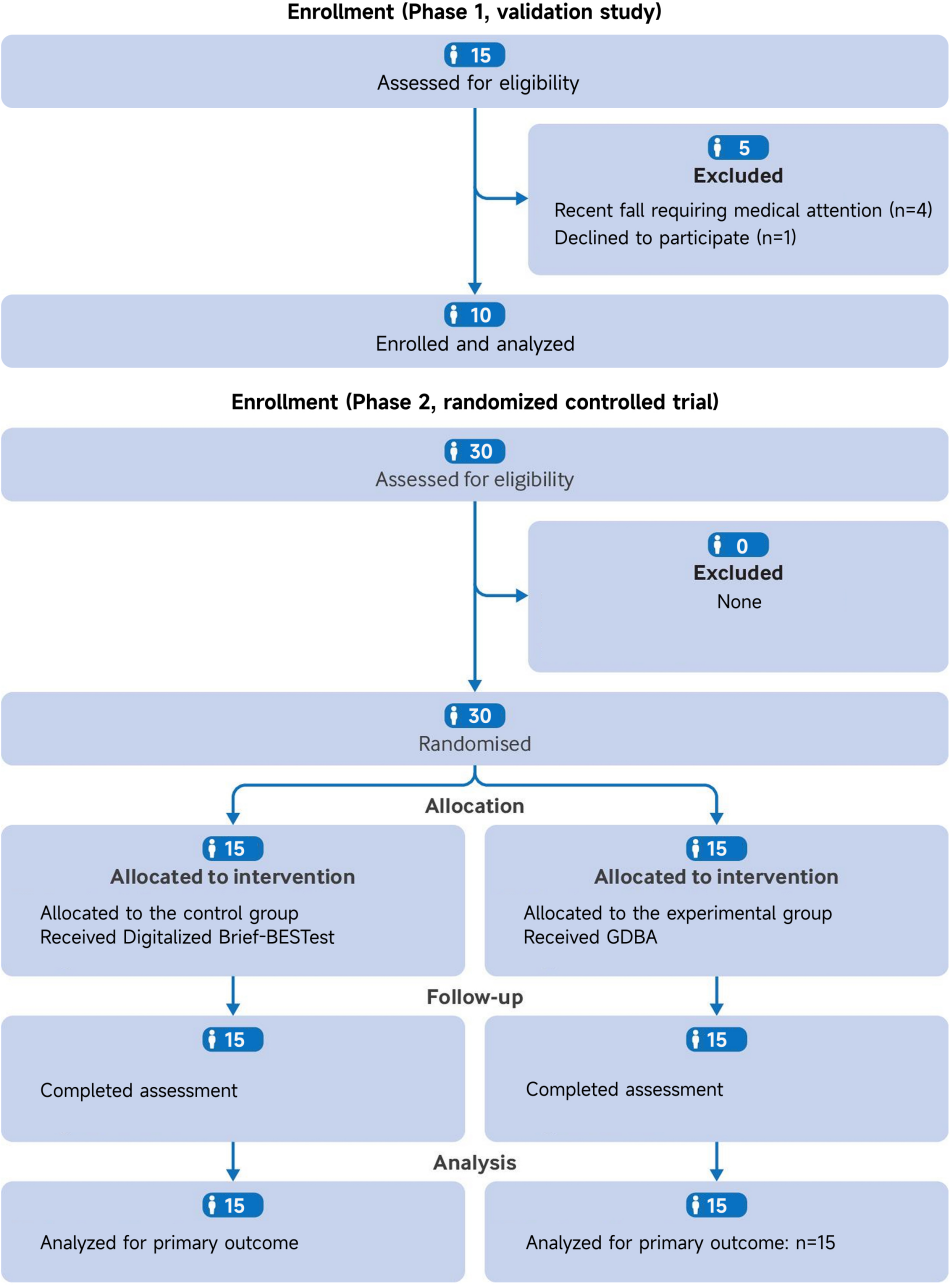
CONSORT (Consolidated Standards of Reporting Trials) flow diagram. Brief-BESTest: Brief Balance Evaluation Systems Test; GDBA: Gamified Digital Balance Assessment;

A total of 30 older adults (age mean 66.7, SD 3.93) were recruited for the study and randomized into 15 in the experimental group and 15 in the control group. The recruited participants consisted of 12 men and 18 women, with 43.34% reporting a history of falls. The BMI (kg/m^2^) is a mean of 26.73 (SD 3.11), with no significant difference between the experimental group and the control group (*P*=.45). All older adults successfully and independently completed the balance tests, with none of the participants missing in the second round of testing. The baseline information is specified in Table 1. Meanwhile, the balance ability for the control group (mean 8.67, SD 4.12) and the experimental group (mean 19.4, SD 3.60; *P*=.61) as well as the balance confidence for the control group (mean 0.72, SD 0.18) and the experimental group (mean 0.84, SD 0.17; *P*=.08) were assessed. Indicating similar balance ability and balance confidence between 2 groups of participants. No harm was documented in the experiment. The primary outcomes were user experience measures (perceived exertion, intrinsic motivation, and intention to continue use). The experiment results are specified in Table 2. The CONSORT flow diagram is specified in Figure 4.

**Table 1.** Baseline demographic and clinical characteristics of community-dwelling older adults participating in Phase 2 (n=30) randomized controlled trial comparing digitalized Brief Balance Evaluation Systems Test (Brief-BESTest; control) versus Gamified Digital Balance Assessment (experimental). Hongqi Community, Shanghai, China, April-May 2025 (N=30).

Characteristic	Total (n=30)	Control group (n=15)	Experimental group (n=15)	*P* value
Age (years), mean (SD)	66.7 (3.93)	65.8 (3.32)	67.6 (4.39)	.21
Gender (female), n(%)	18 (60)	10 (66.67)	8 (53.33)	—^a^
History of falls, n (%)	13 (43.34)	7 (46.67%)	6 (40)	—
BMI (kg/m2), mean (SD)	26.75 (3.11)	26.58 (3.19)	26.92 (3.03)	.45
Balance ability, mean (SD)	19.04 (3.86)	18.67 (4.12)	19.4 (3.60)	.61
Balance confidence, mean (SD)	0.78 (0.18)	0.72 (0.18)	0.84 (0.17)	.08

a Not applicable.

**Table 2.** Comparison of balance performance and user experience outcomes between the control group (digitalized Brief Balance Evaluation Systems Test [Brief-BESTest], n=15) and the experimental group (Gamified Digital Balance Assessment, n=15) in the Phase 2 randomized controlled trial among community-dwelling older adults. Hongqi Community, Shanghai, China, April-May 2025 (N=30).

Outcome measure	Control group (n=15), mean (SD)	Experimental group (n=15), mean (SD)	Mean difference (95% CI)	Cohen *d*	*P* value
Perceived exertion	12.60 (2.75)	9.93 (2.12)	−2.67 (−4.60 to −0.74)	−1.08	.01
Enjoyment and interest	2.73 (1.28)	4.26 (1.33)	1.53 (0.44 to 2.62)	1.17	.009
Perceived competence	3.13 (1.19)	4.27 (1.33)	1.14 (0.19 to 2.09)	0.89	.02
Pressure and tension	3.40 (1.12)	4.13 (1.19)	−0.73 (−1.60 to 0.14)	0.63	.09
Intention to continue use	3.07 (1.28)	4.73 (1.39)	1.66 (0.70 to 2.62)	1.25	<.01

### Perceived Exertion

The study examined the perceived exertion level of participants using the Borg RPE scale. The control group reported a mean perceived exertion of 12.60 (SD 2.75; 95% CI 11.1‐14.1), while the experimental group reported 9.93 (SD 2.12; 95% CI 8.8‐11.1). Participants using the GDBA had significantly lower perceived exertion levels compared to those using the digitalized Brief-BESTest (MD –2.67; 95% CI –4.60 to –0.74; *P*=.01; Cohen *d*=–1.08), representing a large effect size.

### Intrinsic Motivation

The IMI subscales were used to measure participants’ enjoyment and interest, perceived competence, and pressure and tension during the testing process.

For perceived enjoyment and interest, the experimental group reported a significantly higher level (mean 4.26, SD 1.33; 95% CI 3.5‐5.0) compared to the control group (mean 2.73, SD 1.28, 95% CI 2.0‐3.5; MD 1.53; 95% CI 0.44-2.62; *P*=.009; Cohen *d*=1.17), indicating a large effect size.

In terms of perceived competence, the experimental group scored higher (mean 4.27, SD 1.33; 95% CI 3.5‐5.0) than the control group (mean 3.13, SD 1.19; 95% CI 2.5‐3.8). The use of the GDBA resulted in a significant improvement in perceived competence (MD 1.14; 95% CI 0.19-2.09; *P*=.02; Cohen *d*=0.89), representing a large effect size.

In the dimension of perceived pressure and tension, the experimental group (mean 4.13, SD 1.19; 95% CI 3.5‐4.8) reported a lower sense of pressure and tension compared with the control group (mean 3.40, SD 1.12; 95% CI 2.8‐4.0), though the difference was not statistically significant (MD –0.73; 95% CI –1.60 to 0.14; *P*=.09; Cohen *d*=0.63).

### Intention to Continue Use

#### Overview

Quantitative ratings revealed that participants in the experimental group reported a significantly higher intention to continue use (mean 4.73, SD 1.39; 95% CI 3.9‐5.5) compared with those in the control group (mean 3.07, SD 1.28; 95% CI 2.3‐3.8). An independent-samples *t* test confirmed this difference was statistically significant (MD 1.66; 95% CI 0.70-2.62; *P*<.01; Cohen *d*=1.25), indicating a large effect size.

Thematic analysis of the interview transcripts identified 2 overarching themes influencing continued use (E=Experimental, C=Control).

#### Theme 1: Motivational Rewards

Twelve of 15 GDBA users (80%) cited real-time feedback as motivating, “Seeing my balance score appear right after each task felt rewarding... It kept me coming back.” (E04, female, 68).

Seven participants noted game-like features triggered competitive motivation, “When I saw ’Excellent Balance!' with a gold star, I smiled... I wanted that on every task.” (E09, female, 71).

In contrast, control participants noted the absence of feedback as demotivating, “I had no idea how I performed until the end. It felt empty.” (C03, female, 67).

#### Theme 2: Perceived Usability

Thirteen GDBA participants (87%) emphasized avatar demonstrations reduced cognitive load, “The 3D character showing exactly what to do was so helpful... I just copied the avatar.” (E01, female, 72).

Ten control participants (67%) cited lack of variety as a barrier, “It’s functional, but boring... There’s nothing interesting about it.” (C05, male, 66).

### Integration of Quantitative and Qualitative Findings

Qualitative interview data provided convergent evidence supporting the quantitative findings. The significantly higher enjoyment scores in the GDBA group (quantitative) were corroborated by participants’ qualitative descriptions of the experience as “fun,” “engaging,” and “motivating.” Specifically, 12 of 15 (80%) GDBA participants mentioned real-time visual feedback as a key source of enjoyment, stating it made the assessment feel “like a game” rather than a clinical test. In contrast, control group participants described the digitalized Brief-BESTest as “straightforward” or “clinical,” with 10 of 15 (67%) stating that the lack of interactive elements made the experience feel “monotonous.”

The quantitative finding of lower perceived exertion in the GDBA group was explained qualitatively by participants’ reports that the visual tutorial and feedback helped them “learning faster on what they should do” and “forget about the effort,” suggesting reduced attention to physical strain. Several GDBA participants explicitly stated, “I didn’t feel tired because I was concentrating on understanding each task.”

The significantly higher intention to continue use (quantitative) was illuminated by qualitative themes emphasizing motivational rewards and perceived usability. As one participant noted, “If I could check my balance regularly and see improvements over time, I would definitely use it every few months.” Conversely, control group participants identified specific barriers to continued use, most frequently citing “hard to understand,” “lack of variety,” and “minimal feedback” as reasons they would not return. This triangulation of quantitative and qualitative data strengthens confidence in the conclusion that gamification elements meaningfully enhance engagement and motivation for digital balance assessment among older adults.

## Discussion

### Principal Findings

This study pursued two prespecified objectives: (1) to validate a computer vision–based, digitalized Brief-BESTest against a clinician-administered reference standard and (2) to test whether a gamified interface improves user experience during balance assessment among community-dwelling older adults without compromising assessment performance. Both hypotheses were supported. Phase 1 demonstrated that automated scoring by the digitalized system was statistically equivalent to expert clinician scoring and showed strong concurrent validity, consistent with the hypothesis of adequate reliability and validity. Phase 2 showed that participants assigned to the GDBA reported more favorable user experience across all primary outcomes—including lower perceived exertion, higher intrinsic motivation, and greater intention to continue use—while balance performance was comparable between the groups.

The validation findings are notable because much of the existing literature on camera-based or remote balance testing has concentrated on single-task paradigms, such as the TUG or sit-to-stand tests [10,16], which provide valuable but limited information about postural control. In contrast, the Brief-BESTest assesses 6 theoretically distinct domains of balance [35,36] and its construct validity and fall discrimination ability have been supported across community and institutionalized populations [37]. Transforming such a multidomain instrument into an automated pipeline provides a more clinically comprehensive approach than single-task paradigms [37]. The observed agreement between automated and clinician scoring is consistent with that OpenPose-based skeletal tracking can produce meaningful kinematic measurements in older adults [28]. While our findings should be interpreted as preliminary, they suggest that under standardized conditions, pose estimation algorithms can capture task-relevant movement features aligned with established scoring rubrics [28]. If replicated in larger samples and varied environments, this approach could help extend standardized balance screening beyond hospital, responding to calls for scalable digital fall risk assessment [8].

The user-experience results of gamification align with previous research that indicates gamified elements can improve engagement, enjoyment, and adherence when appropriately tailored to seniors’ preferences and capabilities [19,20]. Systematic reviews have concluded that gamification can enhance engagement, enjoyment, and adherence when appropriately tailored to older users [38]. Our findings extend this body of work by focusing on assessment rather than intervention [39]. This distinction matters because the real-world value of screening and monitoring systems depends on repeated voluntary engagement over time, not only on momentary performance [40]. In assessment settings, older adults may also experience evaluative threat or worry about functional decline, which can reduce engagement and distort user experience [41,42]. The observed improvements in enjoyment, perceived competence, and intention to continue use align with self-determination theory, which posits that supporting competence and autonomy enhances intrinsic motivation [29]. Real-time feedback and avatar demonstrations likely reduced uncertainty and clarified task expectations [43], consistent with senior technology acceptance models emphasizing perceived ease of use and perceived usefulness as determinants of adoption [44]. Qualitative data reinforced these interpretations: participants frequently described the feedback as “rewarding” and the avatar guidance as cognitively supportive, echoing findings that interactive and feedback-rich systems can improve engagement among older adults [21]. The reduction in perceived exertion in the gamified group warrants consideration. Theories of attentional focus suggest that engaging, structured feedback may redirect attention away from bodily strain signals, thereby altering subjective effort perception [38]. Yet, we did not observe significant decreased pressure or tension associated with gamified features, addressing concerns that competitive or evaluative elements may heighten anxiety in older populations [21,45].

Taken together, these findings have practical implications for community screening. Measurement validity is necessary but not sufficient for real-world impact; if older adults experience assessments as confusing or effortful, uptake and repeat use will be limited. By integrating a clinically grounded instrument with older adult–tailored interaction design, systems like GDBA may increase willingness to engage in periodic screening, supporting earlier risk identification and more timely connection to fall prevention services.

### Limitations and Future Directions

Despite these promising outcomes, this study has several limitations that also suggest directions for future research. First, the single-session design precludes conclusions about long-term adherence, learning effects, and sustained motivation; longitudinal randomized trials are needed to determine whether early engagement with GDBA leads to habitual use and clinically meaningful outcomes such as reduced falls, improved independence, and lower health care use. Second, participants were relatively healthy, community-dwelling older adults from one urban district in Shanghai, limiting generalizability to frailer, rural, or digitally inexperienced populations. Future work should recruit more diverse samples and incorporate caregiver-assisted or adaptive onboarding to enhance accessibility. Third, validation occurred under optimal indoor conditions, which may not reflect home environments; subsequent studies should test system robustness under variable lighting, space, and camera angles and develop adaptive algorithms to improve accuracy. Fourth, the small Phase 1 sample and possible observer effects from the physiotherapist’s presence warrant replication in larger, more naturalistic settings. The use of a single primary physiotherapist may also have introduced assessor bias, despite strong interrater reliability on 20% of cases; future studies should use multiple assessors to verify scoring generalizability. Fifth, qualitative limitations include brief interviews, lack of interviewer blinding, and no member checking; future work should use longitudinal interviews, blinded interviewers, and participant validation. Finally, the study did not examine clinical end points or engagement mechanisms; mixed methods research should explore how gamification influences motivation and behavior change and assess cost-effectiveness, feasibility, and adaptive designs to sustain engagement and expand applicability. In addition, although the study protocol was approved by the institutional review board before recruitment, the trial was registered retrospectively at ClinicalTrials.gov after participant enrollment had begun. This may raise concerns about selective reporting. We mitigated this limitation by ensuring that the registered information, the institutional review board–approved protocol, and the outcomes and analyses reported in this paper are fully aligned, and we commit to prospective registration for all future trials of this system.

### Conclusions

This study demonstrates the feasibility of integrating digital assessment with human-centered motivational design in a community-oriented geriatric context. The key contribution to the field lies in providing empirical evidence that evidence-based gamification design can significantly enhance older adults’ intrinsic motivation, reduce perceived physical effort, and increase intention for continued use without inducing performance anxiety or cognitive burden, thereby validating theoretical frameworks for practical application in aging populations. More broadly, the findings suggest that validity and engagement should be treated as co-equal design targets in digital health assessment systems, and gamification serves as an effective tool in enhancing user experience and engagement. In terms of real-world implications, the GDBA represents a scalable, cost-effective technology with the potential to extend accessible balance screening to underserved populations with limited clinical access (particularly in rural or resource-limited areas) and reduce health care use and fall-related morbidity through proactive community-based monitoring. If replicated in larger, prospectively registered, multienvironment studies with longitudinal follow-up, such systems could enable scalable community screening, earlier detection of balance decline, and more proactive fall-prevention referral pathways. Conceptually, this work proposes a translational framework: preserve the clinical assessment criteria in a digital format, validate against expert standards, and optimize experiential design to reduce burden and sustain voluntary engagement among older adults.

## Supplementary material

10.2196/80747 Checklist 1 CONSORT-eHEALTH checklist (V 1.6.1).

## Abbreviations

ABC: Activities-specific Balance Confidence scale
Brief-BESTest: Brief Balance Evaluation Systems Test
CONSORT: Consolidated Standards of Reporting Trials
EVA: ethylene-vinyl acetate
GDBA: Gamified Digital Balance Assessment
ICC: intraclass correlation coefficient
ICMJE: International Committee of Medical Journal Editors
IMI: Intrinsic Motivation Inventory
JARS: Journal Article Reporting Standards
MD: mean difference
RCT: randomized controlled trial
RPE: Borg Rating of Perceived Exertion scale
TUG: Timed Up and Go test
